# Sepsis plasma-derived exosomal miR-1-3p induces endothelial cell dysfunction by targeting SERP1

**DOI:** 10.1042/CS20200573

**Published:** 2021-01-27

**Authors:** Min Gao, Tianyi Yu, Dan Liu, Yan Shi, Peilang Yang, Jie Zhang, Jizhuang Wang, Yan Liu, Xiong Zhang

**Affiliations:** Department of Burns, Ruijin Hospital Affliated to Shanghai Jiao Tong University School of Medicine, Shanghai, China

**Keywords:** endothelial cell dysfunction, exosomal miR-1-3p, sepsis-induced lung injury, SERP1

## Abstract

Acute lung injury (ALI) is the leading cause of death in sepsis patients. Exosomes participate in the occurrence and development of ALI by regulating endothelial cell inflammatory response, oxidative stress and apoptosis, causing serious pulmonary vascular leakage and interstitial edema. The current study investigated the effect of exosomal miRNAs on endothelial cells during sepsis. We found a significant increase in miR-1-3p expression in cecal ligation and puncture (CLP) rats exosomes sequencing and sepsis patients’ exosomes, and lipopolysaccharide (LPS)-stimulated human umbilical vein endothelial cells (HUVECs) *in vitro*. However, the specific biological function of miR-1-3p in ALI remains unknown. Therefore, mimics or inhibitors of miR-1-3p were transfected to modulate its expression in HUVECs. Cell proliferation, apoptosis, contraction, permeability, and membrane injury were examined via cell counting kit-8 (CCK-8), flow cytometry, phalloidin staining, Transwell assay, lactate dehydrogenase (LDH) activity, and Western blotting. The miR-1-3p target gene was predicted with miRNA-related databases and validated by luciferase reporter. Target gene expression was blocked by siRNA to explore the underlying mechanisms. The results illustrated increased miR-1-3p and decreased stress-associated endoplasmic reticulum protein 1 (SERP1) expression both *in vivo* and *in vitro*. SERP1 was a direct target gene of miR-1-3p. Up-regulated miR-1-3p inhibits cell proliferation, promotes apoptosis and cytoskeleton contraction, increases monolayer endothelial cell permeability and membrane injury by targeting SERP1, which leads to dysfunction of endothelial cells and weakens vascular barrier function involved in the development of ALI. MiR-1-3p and SERP1 may be promising therapeutic candidates for sepsis-induced lung injury.

## Introduction

Sepsis is a common complication in patients with serious burns, trauma, or infection that causes life-threatening multiple organ dysfunction syndrome (MODS), including coagulopathy, acute lung injury (ALI), liver damage, and renal failure [1]. Among the major organs, the lungs are not only commonly involved but also one of the most vulnerable organs subjected to serious damage [2]. Patients with sepsis-induced ALI are reported to have more severe organ dysfunction and higher in-hospital mortality than non-sepsis-induced ALI [3]. Endothelial cells constitute the first line of defense in the pathological process of sepsis-induced lung injury, which is the earliest change in effector cells undergoing cell contraction, apoptosis, and membrane destruction, leading to increased leakage of pulmonary vessels and acute lung edema [4].

Therefore, protection of pulmonary vascular endothelial cells to maintain the integrity of their structure and function is particularly crucial when sepsis occurs, which has important clinical significance for reducing mortality.

Increasing studies have revealed that exosomes are involved in sepsis, which circulates along with body fluid to deliver messages between neighboring and distant cell communication [5]. Exosomes are extracellular vesicles secreted by nearly all mammalian cell types with a diameter of approximately 30–200 nm and a cup-shaped bilayer membrane structure [6]. Exosomes carry an important cargo of proteins, nucleic acids (mRNA/miRNA), and lipids that are related to the species of parental cells and deliver them to recipient cells [7], exerting an important role in immune processes, tissue repair, and cancer [8]. The most widely studied cargo of exosomes are miRNAs, which are non-coding single-stranded small RNAs with a length of approximately 20–22 nucleotides [9]. During the development of sepsis, the quality and quantity of circulated exosomal miRNAs changes due to immune stress. Abou El-Khier et al. studied the role of miR-122 as a specific early diagnostic and prognostic biomarker for sepsis [10]. Nearly 46 miRNAs were significantly up-regulated after stimulating lung tissue of mice with lipopolysaccharide (LPS), indicating that miRNAs are involved in the development of lung injury [11]. MiR-126 is one of the most abundantly expressed miRNAs in endothelial cells. Its depletion contributes to impaired vascular growth and increased permeability [12]. MiRNA-1246 mediates LPS-induced endothelial cell apoptosis through targeting angiotensin-converting enzyme 2 (ACE-2); elimination of miR-1246 relieves inflammation and neutrophil infiltration in lung tissue [13]. MiR-539-5p was also reported to attenuate apoptosis and pro-inflammatory cytokine production during ALI by down-regulating the expression of Rho-associated protein kinase 1 (ROCK1) in pulmonary microvascular endothelial cells [14]. Many studies have verified that miRNAs play a key role in homeostasis of vascular endothelial cells by regulating cell stress or inflammatory response.

To study the characteristics of miRNA expression profiles in exosomes of sepsis rats, a cecal ligation and puncture (CLP) model was used in the current research. Exosomes were isolated from plasma for future miRNA sequencing analysis, and miR-1-3p was highly expressed in sepsis rat-derived exosomes.

However, few studies have explored the biofunction of miR-1-3p in ALI. Considering the crucial role of endothelial cell injury in the pathogenesis of sepsis-induced ALI, we speculated that miR-1-3p carried by circulating exosomes during sepsis may be taken up by pulmonary vascular endothelial cells and contribute to damaged endothelial cell function and increased permeability of pulmonary vessels. *In vitro*, LPS-stimulated human umbilical vein endothelial cells (HUVECs) were used to explore the effect of miR-1-3p on endothelial cell function. Our results suggested that elevated miR-1-3p induces endothelial cell injury and dysfunction by inhibiting proliferation and increasing apoptosis, monolayer cell permeability, and membrane injury by targeting stress-associated ER protein 1 (SERP1). MiR-1-3p and its target gene *SERP1* could be intervention targets for lung injury in sepsis.

## Materials and methods

### Animal model and procedure

#### Animals

A total of 42 6–8-week-old male SD rats weighing 100–130 g were purchased from Shanghai Experimental Animal Center by the Department of Animal Science at Shanghai Jiao Tong University School of Medicine (SJTUSM). The feeding environment was specific pathogen-free (SPF) at a temperature of 22°C, humidity of 50–60%, and 12-h alternating light and dark cycle. The rats were allowed free water and food. The experiment was conducted 1 week after the rats adapted to the new environment. All of the experimental protocols were approved by SJTUSM Institutional Animal Care and Use Committee, and the animal experiments were conducted in Animal Center of SJTUSM.

#### CLP for sepsis model

The rats were anesthetized via an intraperitoneal injection of 4% pentobarbital (0.2 ml/kg). The abdomen was shaved and disinfected with alcohol. The cecum after laparotomy, and a ligature was performed on the first one of the cecum with a non-absorbable thread. Two pairs of holes were produced with a 50-ml syringe needle. Part of the intestinal contents were squeezed out, returned to the abdominal cavity, and the wound was closed. The sham group was treated with open and closed abdomens, without CLP. The miR-1-3p antagomir (Ribo, 100 nmol in 20 μl PBS) was locally trachea-administrated to the CLP rat model. With LPS (Sigma, 100 μg in 20 μl PBS). The pain was relieved via a subcutaneous injection of bupivacaine. The rats were anesthetized by intraperitoneal injection of 4% pentobarbital and then killed by cervical dislocation 12 h after surgery. The abdominal aorta blood, intestine, and lung tissue were collected. The plasma endotoxin level was tested with a Chromogenic Endpoint Tachypleus Amebocyte Lysate kit (XF-95301) and exosomes were extracted using an ExoQuick Exosome Precipitation kit (SBI, Cat.# ExoQ5A-1) following the users’ manuals. The wet and dry weight of the left lower lung was measured 24 h later to calculate the wet/dry ratio. The left lung tissue was fixed with formalin or glutaraldehyde for paraffin section and electron microscope observation. The right lung tissue and intestine was stored in −80°C refrigerator for further RNA or protein extraction.

### Human blood samples

Three blood samples of sepsis patients and healthy controls were collected. Ethical approval was from the independent ethics committee of SJTUSM (Number: 2016- 105-T54). The patients were diagnosed with sepsis in the intensive care unit (ICU) of Ruijin Hospital (age 30–60 years). Immediately after diagnosis, plasma from 2–3 ml of blood was obtained and stored in a −80°C refrigerator for further exosomes extraction (Exosome Precipitation kit, SBI, Cat. # ExoQ5A-1). Control group plasma was provided by the healthy individuals (age 30–60 years). Written informed consent was obtained from all of the volunteers.

### Cell culture and treatment

HUVECs were cultured with DMEM high-glucose medium supplied with 10% fetal bovine serum (FBS), 100 U/ml of penicillin and streptomycin (Gibco, Gaithersburg, MD, U.S.A.) in a cell incubator at 37°C, and 5% CO_2_. Exosomes were extracted from plasma of 2 ml aorta blood of CLP or Sham rats using ExoQuick Exosome Precipitation kit (SBI), and then resuspended in 50 μl sterile PBS (Sham-Exo, CLP-Exo). The cells were planted in six-well plate with 2 ml culture medium per well, and treated with 50 μl exosomes (Sham-Exo/CLP-Exo) or LPS, Sigma–Aldrich, St. Louis, MO, U.S.A.) for miR-1-3p detection. The HUVECs were transfected with miR-1-3p mimics, inhibitor, and negative control (NC) (Sangon, Shanghai, China) to up-regulate or down-regulate miR-1-3p expression, respectively. The related sequences were shown in Table 1.

**Table 1:** MiR-1-3p-related sequences

Name	Sequence 5′–3′
Human miR-1-3p mimics	UGGAAUGUAAAGAAGUAUGUAUACAUACUUCUUUACAUUCCAUU
Human miR-1-3p inhibitor	AUACAUACUUCUUUACAUUCCA
NC	Sense: UUCUUCGAACGUGUCACGUTT
	Antisense: ACGUGACACGUUCGGAGAATT

Then si-001, si-002, and si003 (si-SERP1) (RiboBio, Guangzhou, Shanghai) were transfected into the HUVECs to select the one that mostly reduced the expression of SERP1 for future studies. SERP1 plasmid was transfected to up-regulate SERP1 expression in HUVECs. Lipofectamine3000 (Invitrogen, Waltham, MA, U.S.A.) was used as transfection reagent. The supernatant was collected in tubes and the cells were harvested with TRIzol (Invitrogen, Waltham, MA, U.S.A.) or RIPA lysis buffer (Thermo Fisher, Waltham, MA, U.S.A.) for RNA or protein extraction 24 h after transfection and LPS stimulation.

### Phalloidin staining

The cells were treated as previously described, washed with PBS three times, fixed for 15 min with 4% paraformaldehyde at 4°C, and then washed with PBS, followed by incubation in 0.5% Triton X-100 solution for 5 min, and then washed again with PBS. The cytoskeleton was stained with phalloidin (CST, Danvers, MA, U.S.A.) in the dark at room temperature for 30 min. The cytoskeleton was washed with PBS three times followed by DAPI (Invitrogen) staining for the nucleus at room temperature for 1 min. The fluorescence images were then observed under a fluorescence microscope.

### Monolayer cell permeability test (BSA-Evans Blue)

Evans Blue dry powder was configured into a 2% stock solution with PBS, filtered, and stored separately. The stock solution was adjusted to a final concentration of 0.67 mg/ml in DMEM medium containing 4% bovine serum albumin (BSA), which was labeled as Evans Blue BSA. The upper layer of a Transwell chamber was planted with 100 μl of cell suspension containing 2 × 10^5^ cells, while the lower chamber was filled with 600 μl of DMEM medium to maintain the liquid balance and cultured in a cell incubator until monolayer cell fusion. Cell transfection and LPS stimulation were conducted in the upper chamber. After 24 h, the liquid in the upper and lower chambers was aspirated, the upper chamber was filled with 100 μl of EBA working solution, while 600 μl of DMEM medium containing 4% BSA was added to the lower chamber and incubated in the cell incubator for 30 min. Then, 200 μl of lower layer solution was added to a 96-well plate and the absorbance was measured at 620 nm with a microplate spectrophotometer (BioTek, Winooski, VT, U.S.A.).

### Lactate dehydrogenase activity assay

Lactate dehydrogenase (LDH) activity in the supernatant of the cells was assessed by an LDH test kit (Jiancheng, Nanjing, China). Briefly, 1 × 10^5^ cells/well were planted in a 96-well plate with the previously described treatment. The supernatant was collected in clean tubes and mixed with reaction buffer provided by the kit following the manufacturer’s instructions. The absorbance at 490 nm was measured with a microplate spectrophotometer (BioTek, U.S.A.). The experiment was repeated three times.

### Real-time polymerase chain reaction

An miRNA real-time PCR kit (RiboBio, Shanghai, China) was used to reverse transcript the RNA into cDNA for miR-1-3p and U6 detection (stem-loop method). For the mRNA expression of SERP1, glucagon-like peptide 1 receptor (GLP1R), and β-actin, cDNA synthesis and PCR amplification were conducted using HiScriptII Q RT SuperMix and ChamQ Universal SYBR qPCR Master Mix kits (Vazyme, Shanghai, China) on an ABI 7500 Real-Time PCR system (Applied Biosystems, Waltham, MA, U.S.A.). The relative gene expression was expressed using the 2^−ΔΔ*C*_T_^ method with β-actin or U6 serving as internal controls. Primers were synthesized by Sangon (Shanghai, China), and the primer sequences were shown in Table 2.

**Table 2:** MiRNA/mRNA primer sequences

Genes	Sequence (5′–3′)
Rat miR-1-3p stem loop primer	GTTGGCTCTGGTGCAGGGTCCGAGGTATTCGCACCAGAGCCAACATACAC
Rat miR-1-3p	Forward-CGACCCTGGAATGTAAAGAAGReverse-GTGCAGGGTCCGAGGT
U6 stem loop primer	GTCGTATCCAGTGCAGGGTCCGAGGTATTCGCACTGGATACGACAAAATA
U6	Forward-AGAGAAGATTAGCATGGCCCCTG
	Reverse-ATCCAGTGCAGGGTCCGAGG
Human SERP1	Forward-CGCCAAGACCTCGAGAAATG
	Reverse-ACTTCACATGCCCATCCTG
Human GLP-1R	Forward-GCTCTGGTTATCGCCTCTG
	Reverse-GTGCTATACATCCACTTCAGGG
Human β-actin	Forward-CCTCGCCTTTGCCGATCC
	Reverse-GGCCATCTCTTGCTCGAAGT

### Western blotting

Protein samples extracted from the cells or lung tissue were measured by a BCA kit (Thermo Fisher, U.S.A.) for protein concentration evaluation. Proteins (20 μg) in each sample were mixed with loading buffer and denatured at 100°C for 5 min, then subjected to SDS/PAGE for electrophoresis. The membranes were incubated with primary antibodies as follows: SERP1 (Abclonal, Wuhan, China), GLP1R (Abclonal, China), caspase-3 (Abcam, Cambridge, MA, U.S.A.), Bcl-2, VEGF, IL-1β, iNOS, phosphorylated myosin light chain (p-MLC), connexin43 (Cx43), phosphorylated focal adhesion kinase (p-FAK397), and β-actin (Cell Signaling Technology, Danvers, MA, U.S.A.). After incubation at 4°C ernight, the membranes were washed with TBST three ti-es and incubated with corresponding secondary antibodies for 2 h at room temperature. Images were detected by an Odyssey Infrared Imaging System (LI-COR, Lincoln, NE, U.S.A.) with enhanced chemiluminescence (Millipore, Burlington, MA, U.S.A.). The grayscale value of each protein signal was compared relative to β-actin.

### Cell proliferation assay by cell counting kit-8

The HUVECs (5 × 10^3^ cells/well) were seeded into 96-well plates for a proliferation test. After cultivation for 16 h, the cells were transfected with small RNA or siRNA as previously described with or without LPS stimulation for 24, 48, and 72 h. The cell proliferation was determined according to the protocols of the Cell Counting Kit-8 (CCK-8) kit (Dojindo, Kumamoto, Japan). The absorbance was measured at 450 nm with a microplate spectrophotometer (BioTek, U.S.A.). The experiment was repeated three times.

### Flow cytometry analysis for apoptosis

The HUVECs were planted in six-well plate and transfected with miR-1-3p mimics/inhibitor/NC or si-SERP1 or SERP1 plasmid with or without LPS stimulation for 24 h. The cells were collected and centrifuged at 1000 rpm for 5 min. The supernatant was discarded and then the cells were washed with PBS twice. The cells were resuspended in 100 μl of 1× Annexin V binding buffer to adjust the cell density at 1 × 10^6^ cells/ml. Annexin V-FITC binding solution and PI were added following the protocols of an Annexin V-FITC/PI Cell Apoptosis Detection Kit (BD Biosciences, East Rutherford, NJ, U.S.A.). Apoptosis analysis was conducted using an LSRII flow cytometer (BD Biosciences, U.S.A.) with CellQuest software to analyze the results (FITC Annexin V^−^/PI^−^ cells were considered apoptotic). The experiment was repeated three times.

### Dual-luciferase reporter assay

MiR-1-3p target genes were predicted using the TargetScan database (www.targetscan.org) and miRDB (www.microRNA.org). The SERP1-3′ untranslated region (UTR) reporter vectors were constructed by Ribo (Guangzhou, China). 293T cells were grown to 50–80% confluence in 12-well plates and co-transfected wild-type (SERP1-WT)/mutated (SERP1-Mut) reporter vectors with miR-1-3p mimics or NC mimics using Lipofectamine 3000. Luciferase assays were conducted 48 h post-transfection using Dual-Luciferase Reporter Assay System (Promega, San Luis Obispo, CA, U.S.A.). Luciferase activity was measured using a luminometer (Promega, U.S.A.) following the manufacturer’s instructions. Each sample was measured in triplicate.

### Statistical analysis

The data were expressed as means ± SD. SPSS21 and GraphPad Prism 7 software were used for the statistical analysis. Significant differences were determined via Student’s *t* test or one- or two-way analysis of variance. *P*<0.05 was considered statistically significant.

## Results

### Exosomal miR-1-3p overexpressed *in vivo* and *in vitro*

**Figure 1:**
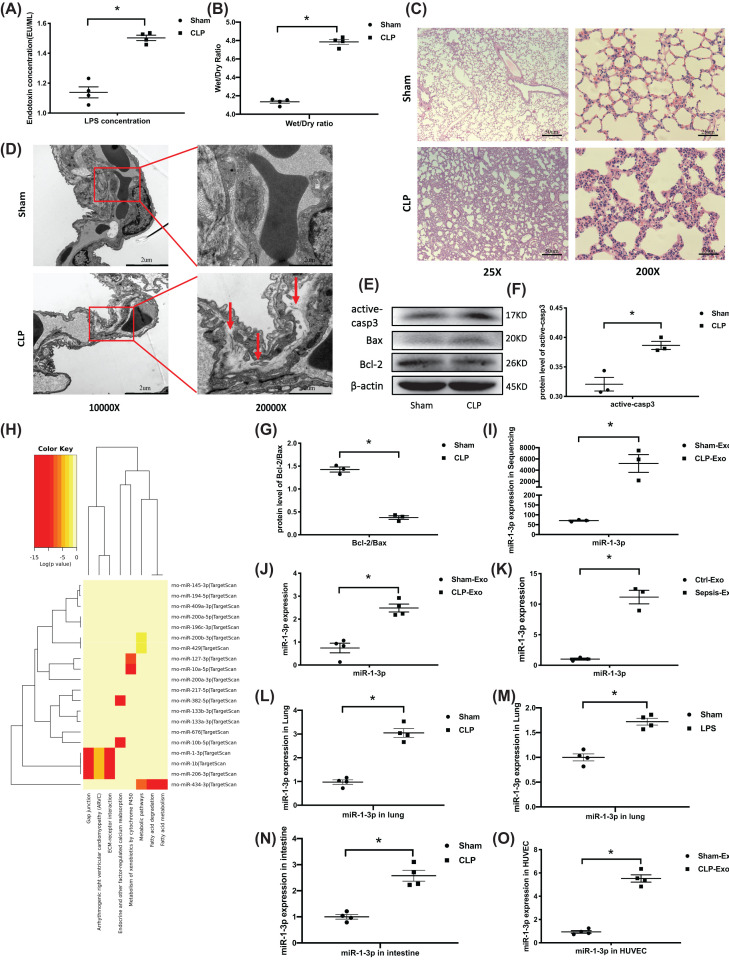
Exosomal miR-1-3p overexpressed *in vivo* and *in vitro* (**A**) Endotoxin level of rat blood was assessed using an endotoxin detection kit 12 h after the CLP model was established (*n*=4, from four individuals). (**B**) The wet and dry weight (24 h) of the left lower lung was recorded and calculated for the wet/dry ratio (*n*=4, from four individuals). (**C**) Representative H&E staining of lung tissue under microscopy observation. The images are representative of four individuals. (**D**) Observation of CLP rat lung by electron microscopy. The images are representative of four individuals. (**E–G**) Western blotting analysis of active-casp3, Bcl-2, and Bax expression in the lung tissue of the CLP model, and β-actin served as an internal reference. The blots are representative of at least three independent experiments with similar results. (**H**) Exosomes were extracted from the plasma of sham and CLP rats by precipitation and then subjected to miRNA sequencing. KEGG analysis of the top 20 most significantly different miRNAs’ pathways. (**I**) MiR-1-3p was significantly expressed in the sequencing of CLP plasma-derived exosomes (CLP-Exo) (*n*=3, from three independent experiments). (**J,K**) RT-PCR to confirm the expression of miR-1-3p in CLP rats and sepsis patients’ (*n*=3, from three independent experiments) plasma-derived exosomes. (**L**) RT-PCR test of miR-1-3p expression in lung tissue of CLP rat (*n*=4, from four independent experiments). (**M**) RT-PCR test of miR-1-3p expression in the lung tissue of LPS administrated rat (*n*=4, from four independent experiments). (**N**) RT-PCR test of miR-1-3p expression in the intestine of CLP rat (*n*=4, from four independent experiments). (**O**) MiR-1-3p expression in Sham-Exo and CLP-Exo (precipitated from 50 μl of plasma) treated HUVECs for 24 h by RT-PCR (*n*=4, from four independent experiments). Data are presented as mean ± SE, *P<0.05. Abbreviation: RT-PCR, real-time polymerase chain reaction.

The endotoxin level of plasma separated from CLP rats was measured. As shown in Figure 1A, the endotoxin concentration in the CLP group was significantly higher than that in the sham group. The wet/dry ratios of the two groups were compared to measure the degree of pulmonary edema. The wet/dry ratio of the lung tissue in the CLP group was 4.79 ± 0.05 and in the normal group was 4.13 ± 0.03, a significant difference was observed between the two groups (Figure 1B). H&E staining showed obvious pulmonary edema with disordered lung structure, thickened alveolar wall, and a large amount of inflammatory cell infiltration in the CLP group compared with the sham group (Figure 1C). The ultrastructure of the lung tissue under an electron microscope showed no visible swelling of the endothelial cells in the sham group.

However, the connection between endothelial cells and basement membranes was damaged by fluid accumulation in the CLP group (Figure 1D, shown by the red arrow). The results confirmed the pulmonary edema in the CLP rats. The expression of apoptosis-related proteins in the lung tissue was evaluated by Western blotting. Pro-apoptotic protein active-casp3 was increased with a decreased Bcl-2/Bax ratio in the CLP group (Figure 1E–G), demonstrating increased apoptosis of the lung tissue in the CLP group.

Exosomes were isolated from CLP rat plasma for further miRNA sequencing. According to a KEGG analysis of the top 20 most significantly different miRNA pathways, the function of the miRNAs was mainly focused on regulating gap junctions, extracellular matrix receptor interactions, and calcium reabsorption (Figure 1H). Among these, miR-1-3p was the most highly expressed miRNA in the CLP-derived exosomes (CLP-Exo), which was approximately 50-times higher than the control group (sham-Exo) (Figure 1I). This was further confirmed by RT- PCR testing of the miR-1-3p expression in CLP rats and sepsis patients’ exosomes (Figure 1J,K), as well as lung tissue of CLP rat and local tracheal LPS administration rat (Figure 1L,M). Considering intestine was also one of the sources of circulated exosomes, real-time polymerase chain reaction (RT-PCR) was taken to demonstrate the increased miR-1-3p in intestine of CLP rat model (Figure 1N). Moreover, the HUVECs also showed significantly increased miR-1-3p after treatment with CLP-Exo for 24 h (Figure 1O). The results indicated that overexpressed circulating exosomal miR-1-3p may have been taken up by vascular endothelial cells during sepsis and were involved in the progress of sepsis-induced lung injury.

### MiR-1-3p regulates proliferation, apoptosis, and related protein expression of HUVECs *in vitro*

**Figure 2:**
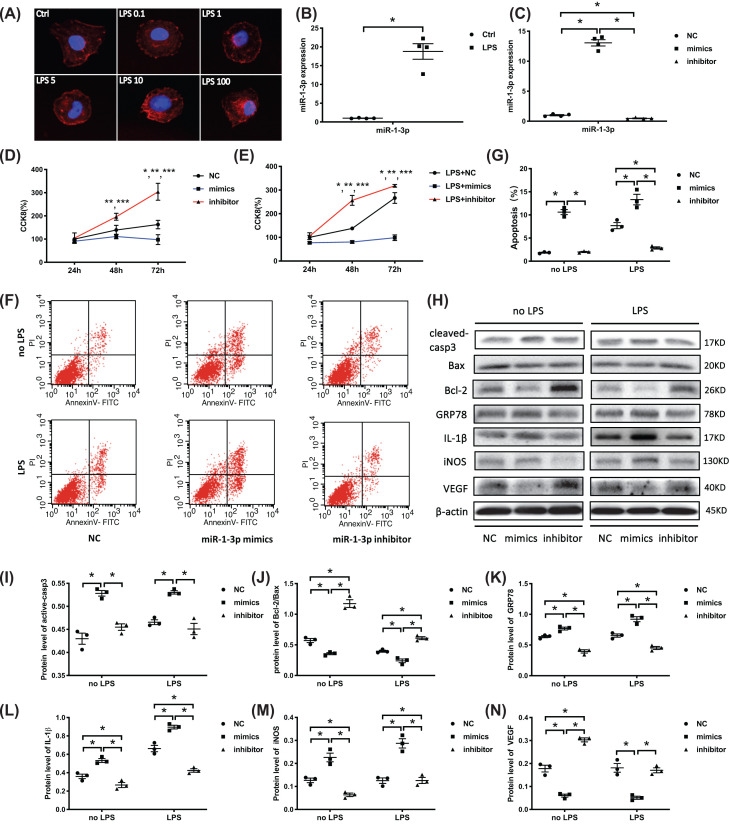
MiR-1-3p regulates proliferation, apoptosis, and related protein expression of HUVECs *in vitro* (**A**) HUVECs were treated with LPS at different concentrations (0.1, 1, 5, 10, and 100 μg/ml). Cytoskeleton F-actin was stained with phalloidin and observed under a fluorescence microscope (400×). (**B**) MiR-1-3p expression analysis of LPS (10 μg/ml) stimulated HUVECs for 24 h by RT-PCR (*n*=4, from four independent experiments). (**C**) MiR-1-3p expression of HUVECs transfected with miR-1-3p mimics, inhibitor, and NC by RT-PCR detection (*n*=4, from four independent experiments). (**D,E**) CCK-8 for cell proliferation tests with or without LPS stimulation (*n*=6, from three independent experiments with duplicate, *P<0.05 in NC vs mimics, **P<0.05 in NC vs inhibitor, ***P<0.05 in mimics vs inhibitor). (**F,G**) Cell apoptosis assay by flow cytometry (*n*=3, from three independent experiments). (**H**–**N**) Western blotting evaluated the expression of apoptosis-related proteins active-casp3, Bcl-2, Bax, ER stress protein GRP78, VEGF, and proinflammatory cytokines IL-1β and iNOS. β-actin served as an internal reference. The blots are representative of at least three independent experiments with similar results. Data are presented as mean ± SE, *P<0.05.

The HUVECs were treated with LPS *in vitro* to simulate damage to vascular endothelial cells during sepsis-induced lung injury. Phalloidin-stained F-actin showed cytoskeleton morphology changes in the HUVECs under different LPS concentrations (mg/l). As shown in Figure 2A, the cytoskeleton changed from a uniform network-like distribution in the cytoplasm to thickened and disordered accumulation along the nucleus, and cell shrinkage was visible as the LPS concentrations increased. Considering the cytotoxicity of high LPS concentrations, 10 mg/l of LPS was used for subsequent experiments. MiR-1-3p was obviously enhanced in the LPS-treated HUVECs at this concentration (Figure 2B).

To investigate the biological function of miR-1-3p on endothelial cells *in vitro*, HUVECs were transfected with miR-1-3p mimics, inhibitor, and NC to increase or block miR-1-3p expression separately. The transfect efficiency was evaluated via RT- PCR (Figure 2C). CCK-8 assays were conducted to observe the influence of miR-1-3p on cell proliferation. Overexpressed miR-1-3p significantly inhibited cell growth, while blocking miR-1-3p promoted proliferation 48 and 72 h after transfection with or without LPS treatment (Figure 2D,E). Flow cytometry found that apoptosis increased in the mimics group and decreased in the inhibitor group (Figure 2F,G).

Western blotting showed increased active-casp3 and a decreased Bcl-2/Bax ratio in the mimics group, further confirming the promotion effect of miR-1-3p on HUVECs apoptosis. Elevated endoplasmic reticulum (ER) stress marker glucose regulator protein 78 (GRP78), inflammatory cytokines IL-1β, and iNOS protein levels, and decreased VEGF expression were also observed in the mimics group by Western blotting (Figure 2H–N), while down-regulating miR-1-3p by inhibitor showed opposite changes with or without LPS stimulation. The results demonstrated that overexpressed miR-1-3p inhibited proliferation and promoted apoptosis as well as inflammatory response of HUVECs, which can be reversed by down-regulating the expression of miR-1-3p.

### MiR-1-3p contributed to increased permeability and HUVECs membrane injury

**Figure 3:**
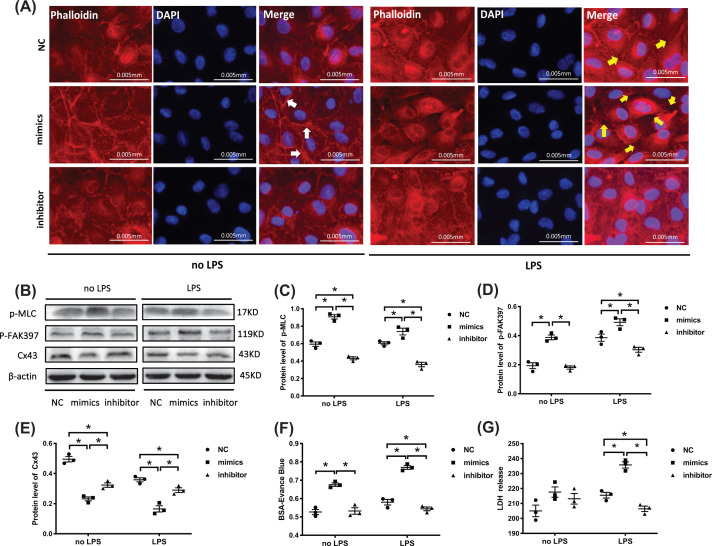
MiR-1-3p contribute to enhanced permeability of HUVECs (**A**) HUVECs cytoskeleton F-actin was stained with phalloidin after miR-1-3p NC/mimics/inhibitor transfection with or without LPS treatment. The images are representative of three experiments with similar results. (**B**–**E**) Western blotting analysis of p-MLC, p-FAK397, and Cx43 expression. β-actin served as an internal reference. The blots are representative of at least three independent experiments with similar results. (**F**) Transwell assays measured the effect of miR-1-3p on monolayer cell permeability by detecting the absorbance of BSA-Evans Blue (*n*=3, from three independent experiments). (**G**) LDH activity detection in the supernatant of transfected cells by an LDH detection kit (*n*=3, from three independent experiments). Data are presented as mean ± SE, *P<0.05.

Phalloidin-stained F-actin was used to observe the HUVECs cytoskeleton with miR-1-3p mimics or inhibitor transfection (Figure 3A). In the absence of LPS stimulation, stress fibers (white arrows) appeared and distributed around the cells when the cytoskeleton contracted in the mimics group. There were no significant changes in the inhibitor group compared with NC group. After LPS stimulation, a slight intercellular space (yellow arrows) was observed in the NC group. An obvious nucleus distribution of the contracted cytoskeleton was found in the mimics group, which resulted in cell shrinkage and more intercellular spaces. However, no apparent intercellular spaces appeared in the inhibitor group.

Cell permeability-related proteins, p-MLC and p-FAK397, increased in the mimics group and decreased in the inhibitor group compared with NC group, while Cx43 presented an opposite pattern under the same conditions (Figure 3B–E). Evans Blue-labeled BSA was used as an indicator to assess changes in the monolayer endothelia cell permeability under miR-1-3p regulation. Under LPS treatment, significantly increased BSA-Evans Blue was detected in the lower chamber of the mimics group, but decreased in the inhibitor group compared with the NC group (Figure 3F). LDH activity in the supernatant of the mimics group visibly increased under LPS stimulation, indicating damage of miR-1-3p to the cell membrane (Figure 3G). These results proved that miR-1-3p overexpression contributes to enhanced monolayer cell permeability and HUVECs membrane injury.

### SERP1 is the target gene of miR-1-3p targets

**Figure 4:**
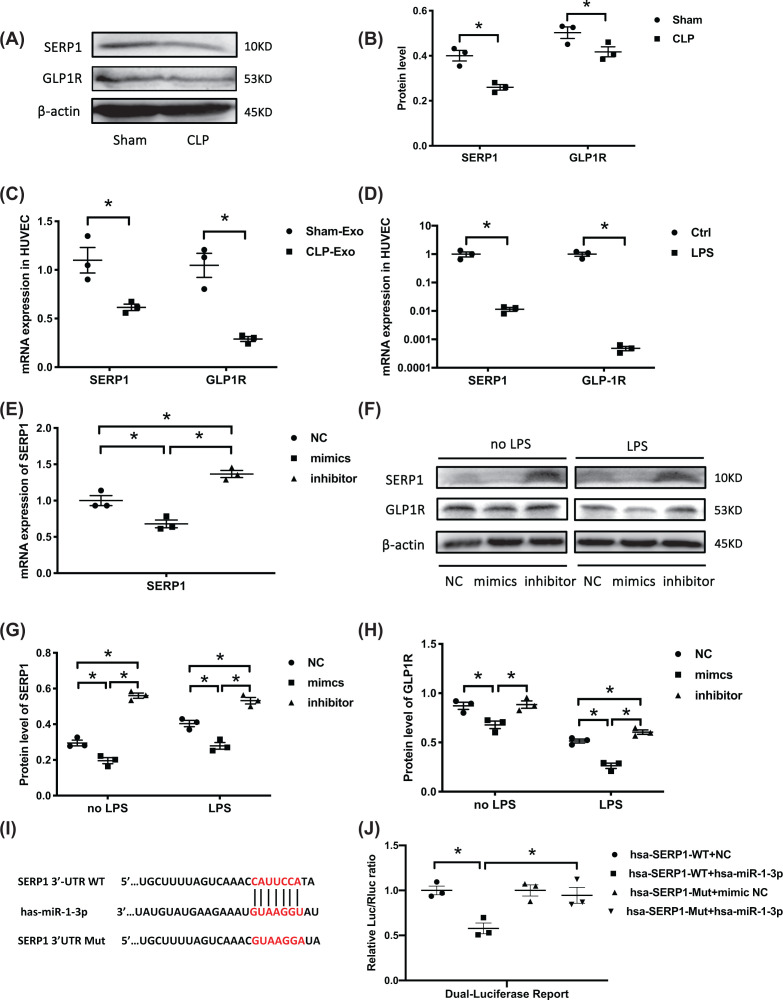
MiR-1-3p directly targets the 3′UTR of SERP1 and inhibits its expression (**A,B**) Western blotting analysis of SERP1 and GLP1R expression in the lung tissue of CLP rats. The blots are representative of at least three independent experiments with similar results. (**C,D**) MRNA expression of SERP1 and GLP1R in CLP-exo and LPS (10 μg/ml) treated HUVECs for 24 h by RT-PCR (*n*=3, from three independent experiments). (**E**) SERP1 mRNA expression under the treatment of miR-1-3p mimics, inhibitor, and NC (*n*=3, from three independent experiments). (**F–H**) Western blotting detection of GLP1R and SERP1 protein levels in HUVECs transfected with miR-1-3p mimics, inhibitor, and NC. β-actin served as an internal reference. The blots are representative of at least three independent experiments with similar results. (**I**) Binding site of SERP1 mRNA 3′-UTR (SERP1-WT) with miR-1-3p and designed SERP1 3′-UTR mutant (SERP1-Mut) sequence. (**J**) Dual-luciferase reporter assay of miR-1-3p and SERP1 mRNA 3′-UTR binding sites (*n*=3, from three independent experiments). Data are presented as mean ± SE, *P<0.05.

To investigate the potential target genes that induced miR-1-3p biofunction, the TargetScan and miRDB databases were used to analyze related potential genes, and SERP1 was identified as a perspective target. SERP1 was reported to regulate cell proliferation and apoptosis as well as cell membrane stability by increasing glycosylation levels of GLP1R [15]. The protein levels of SERP1 and GLP1R were reduced in the CLP rats’ lung tissue compared with the sham group (Figure 4A,B). The mRNA expression of SERP1 and GLP1R was also significantly decreased in the CLP-Exo- (Figure 4C) and LPS- (Figure 4D) treated HUVECs.

HUVECs were transfected with mimics and inhibitor to explore the regulation effect of miR-1-3p on SERP1. RT-PCR showed repressed SERP1 mRNA in the mimics group and increased expression under inhibitor treatment (Figure 4E). Western blotting exhibited a consistent trend in SERP1 and GLP1R proteins with mRNA expression (Figure 4F–H). In addition, luciferase reporter of wild-type (WT) plasmid containing 3′UTR sequence of SERP1 and its mutant (Mut) plasmid were transfected to cells with miR-1-3p or NC. Dual-luciferase assay demonstrated reduced fluorescence ratios in the hsa-SERP1-WT + hsa-miR-1-3p group, but the mutant 3′-UTR of SERP1 group (SERP1-Mut) showed no changes (Figure 4I,J). Our results verified that SERP1 is a direct negatively regulated target gene of miR-1-3p.

### Down-regulated SERP1 inhibits proliferation and promotes apoptosis of HUVECs

**Figure 5:**
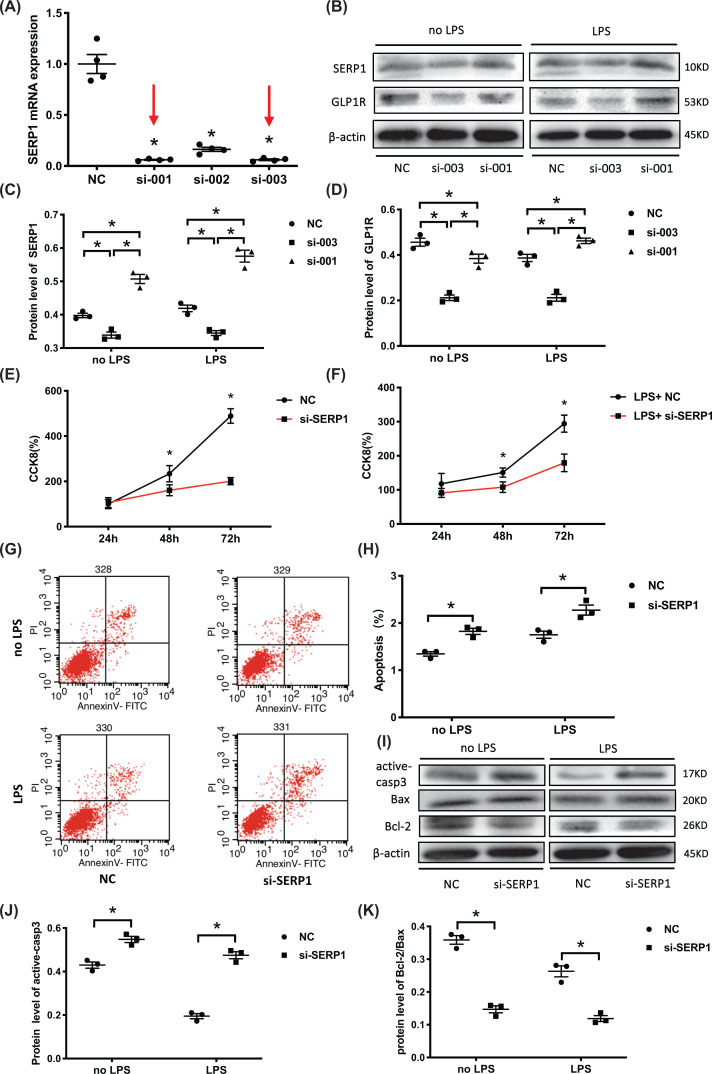
Down-regulated SERP1 inhibits proliferation and promotes apoptosis of HUVECs (**A**) HUVECs were transfected with SERP1 siRNA (si-001, si-002, and si-003), and mRNA expression of SERP1 was assessed by RT-PCR to select the most efficient siRNA (*n*=4, from four independent experiments). (**B**–**D**) Western blotting for SERP1 and GLP1R protein levels under siRNA transfection. Si-003 was the most efficient siRNA. The blots are representative of at least three independent experiments with similar results. (**E,F**) Effect of decreased SERP1 on HUVECs proliferation with or without LPS stimulation (*n*=6, from three independent experiments with duplicate). (**G,H**) Effect of decreased SERP1 on HUVEC apoptosis with or without LPS stimulation (*n*=3, from three independent experiments). (**I–K**) Western blotting analysis of apoptosis-related proteins active-casp3, Bcl-2, and Bax expression. The blots are representative of at least three independent experiments with similar results. β-actin served as an internal reference. Data are presented as mean ± SE, *P<0.05.

To determine whether the effects of miR-1-3p on the HUVECs were induced by SERP1, siRNAs (si-001, si-002, and si-003) were used to block SERP1 mRNA expression. Combining the results of RT-PCR with Western blotting, si-003 was the most efficient siRNA for SERP1 silencing (Figure 5A–D). The cells transfected with si- SERP1 (si-003) had significantly decreased proliferation after 48 and 72 h compared with the NC group with or without LPS present (Figure 5E,F). Blocking SERP1 expression also contributed to enhanced apoptosis of HUVECs detected by flow cytometry (Figure 5G,H), with increased active-casp3 and decreased Bcl-2/Bax ratios (Figure 5I–K). Taken together, the results demonstrated that down-regulated SERP1 inhibited HUVECs proliferation and promoted apoptosis *in vitro*.

### SERP1 induces HUVECs dysfunction by prompting permeability, membrane injury, and inflammatory response

**Figure 6:**
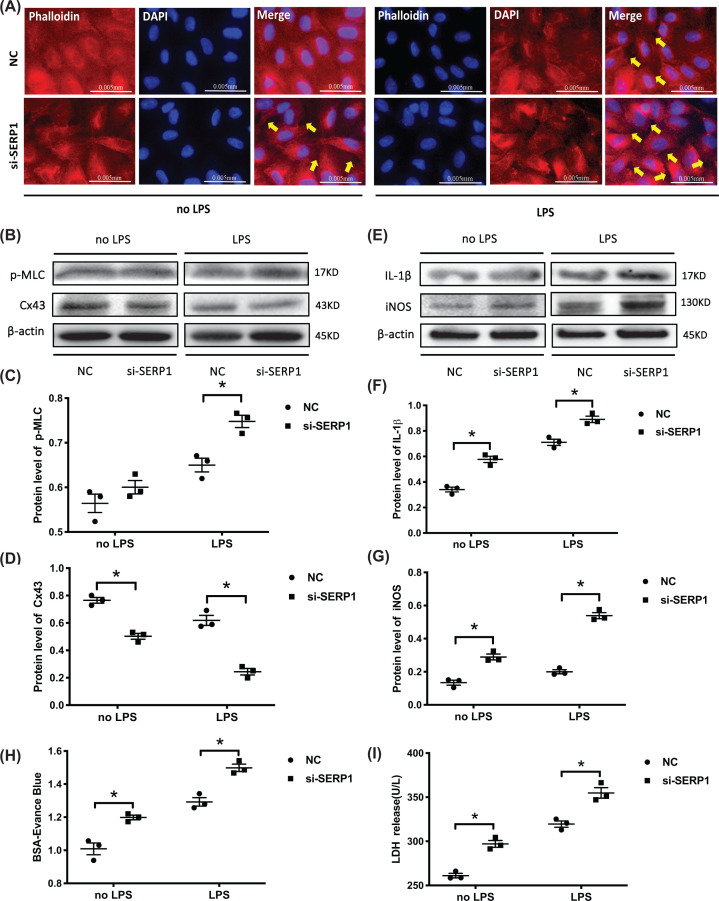
SERP1 induces HUVECs dysfunction by prompting permeability, membrane injury, and inflammatory response (**A**) HUVECs cytoskeleton F-actin was stained with phalloidin after transfection with si-SERP1 with or without LPS treatment (400×). The images are representative of three experiments with similar results. (**B–D**) Western blotting detection of HUVECs permeability related p-MLC and Cx43 expression after transfection with si-SERP1. The blots are representative of at least three independent experiments with similar results. (**E**–**G**) Western blotting detection of inflammatory cytokine IL-1β and iNOS expression after transfection with si-SERP1 in HUVECs. The blots are representative of at least three independent experiments with similar results. (**H**) Transwell assays measured the effect of SERP1 on monolayer cell permeability by detecting the absorbance of BSA-Evans Blue (*n*=3, from three independent experiments). (**I**) LDH activity analysis in the supernatant of si-SERP1 transfected HUVECs by an LDH detection kit (*n*=3, from three independent experiments). β-actin served as an internal reference. Data are presented as mean ± SE, *P<0.05.

The effect of SERP1 on cell contraction was investigated by staining cytoskeleton F-actin with phalloidin. With or without LPS stimulation, the SERP1 elimination group displayed an obvious cytoskeleton contraction and intercellular space (Figure 6A). Furthermore, the protein level of p-MLC increased while Cx43 declined after silencing SERP1 expression, accompanied by elevated proinflammatory factors IL-1β and iNOS (Figure 6B–G). In addition, Transwell assay showed increased BSA-Evans Blue leakage after SERP1 down-regulation (Figure 6H). LDH activity also increased in the supernatant of si-SERP1 group (Figure 6C). These results revealed that SERP1 inhibition caused increased cell contraction, inflammatory response, monolayer endothelial cell permeability and membrane damage, which demonstrated a similar effect on the miR-1-3p mimics on HUVECs dysfunction.

### SERP1 overexpression protects lung tissue and HUVECs from deleterious effects of miR-1-3p

**Figure 7:**
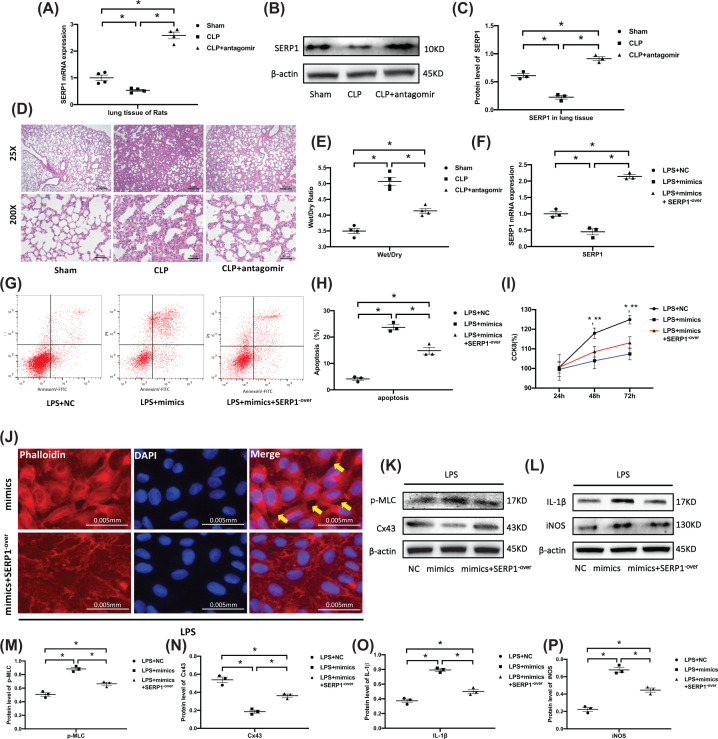
SERP1 overexpression protects lung tissue and HUVECs from deleterious effects of miR-1-3p (**A**) SERP1 mRNA expression in the lung tissue after miR-1-3p antagomir was locally trachea-administrated to the CLP rat model (*n*=4, from four individuals). (**B,C**) Western blotting analysis of SERP1 expression in the lung tissue, with β-actin served as an internal reference. The blots are representative of at least three independent experiments with similar results. (**D**) H&E staining of lung tissue under microscopic observation. The images are representative of three experiments with similar results. (**E**) The wet/dry ratio of the left lower lung in each group (*n*=4, from four individuals). (**F**) HUVECs were transfected with SERP1 plasmid (SERP1^−over^) and/or mimics, and mRNA expression of SERP1 was assessed by RT-PCR to verify the transfection efficiency (*n*=3, from three independent experiments). (**G,H**) Flow cytometry analysis of apoptosis after SERP1 overexpression (*n*=3, from three independent experiments). (**I**) Effect of SERP1 overexpression on HUVEC proliferation was assessed by CCK8 (*n*=6, from three independent experiments with duplicate). *P<0.05 in LPS+NC vs LPS+mimics, **P<0.05 in LPS+NC vs LPS+mimics+SERP1^−over^. (**J**) HUVECs cytoskeleton F-actin was stained with phalloidin after transfecting with SERP1 plasmid (SERP1^−over^) and/or mimics under LPS treatment (fluorescence microscope, 400×). The images are representative of three experiments with similar results. (**K,M,N**) Western blotting analysis of permeability related p-MLC and Cx43 expression after transfecting with SERP1 plasmid (SERP1^−over^) and/or mimics under LPS treatment. β-actin served as an internal reference. The blots are representative of at least three independent experiments with similar results. (**L,O,P**) Western blotting detection of inflammatory cytokine IL-1β and iNOS expression after transfection with SERP1 plasmid (SERP1^−^ over) and/or mimics under LPS situation. The blots are representative of at least three independent experiments with similar results. β-actin served as an internal reference.

To further explore if miR-1-3p is implicated in the occurrence of lung edema, miR-1-3p antagomir was locally trachea-administrated to the CLP rat model. The mRNA and protein expression of target gene SERP1 was increased in CLP+antagomir group, which verified the effect of miR-1-3p antagomir (Figure 7A–C). H&E staining showed obvious relieved pulmonary edema and inflammatory cell infiltration in the CLP+antagomir group compared with the CLP group (Figure 7D). The wet/dry ratio of the lung tissue in CLP group was significantly decreased after miR-1-3p antagomir application (Figure 7E). The SERP1 plasmid was used to up-regulate SERP1 mRNA expression in HUVECs under treatment of miR-1-3p mimics (Figure 7F). Flow cytometry showed that after overexpression of SERP1 in mimics group, the cell apoptosis was significantly reduced (Figure 7G,H). While CCK-8 assays showed no statistical difference between miR-1-3p mimics group and mimics+SERP1^−over^ group at 48 h and 72 h time points under LPS treatment, even though SERP1 overexpression has slightly improved miR-1-3p’s inhibition effect on cell proliferation (Figure 7I). Up-regulated SERP1 also improves intercellular space caused by miR-1-3p mimics in the environment of LPS (Figure 7J). Western blotting showed declined p-MLC and increased Cx43 protein level after SERP1 overexpression in miR-1-3p mimics group (Figure 7K,M,N), along with elevated proinflammatory factors IL-1β and iNOS (Figure 7L,O,P). The results above indicated that miR-1-3p is implicated in lung edema of CLP rats and SERP1 overexpression protects against deleterious effects of miR-1-3p in apoptosis, cell contraction, and inflammation *in vitro*.

## Discussion

Sepsis is an uncontrolled and disordered regulation of immune response and is one of the leading causes of death in ICU patients and an extremely heavy financial burden worldwide [16]. Various bioactive ingredients released into the blood during endotoxin stimulation contribute to the occurrence and progression of sepsis [17].

Among these, exosomes play an important role in mediating cell damage or repair by passing certain cargo including miRNAs into recipient cells [18]. MiRNAs are abundantly expressed in eukaryotes and have an extensive regulatory effects in information exchange, participating in cell growth, proliferation, apoptosis, metabolism, tumor, and infection [19,20]. Further investigation revealed that miRNAs might regulate apoptosis or inflammation response during ALI by targeting specific proteins or regulating related pathways [21]. MiR-146a activated the TLR4/NF-κB signaling pathway by targeting tumor necrosis factor (TNF) receptor-associated factor 6 (TRAF6) or interleukin-1 receptor associated kinase 1 (IRAK1), which resulted in the release of numerous apoptotic factors, such as interleukin-1 and TNF, leading to amplified inflammatory cascade and ultimately the occurrence of ALI/ARDS [22]. Increased miR-486-5p was reported to induce cell apoptosis and promote ALI progress by targeting OTU domain containing 7B (OTUD7B) [23]. Some protective exosomes maintain homeostasis of the internal environment by participating in self-protection mechanisms under disease conditions [24]. Endothelial progenitor cell-derived exosomes inhibit high-mobility group protein B1 (HMGB1) by overexpressing miR-126, which targets phosphoinositide 3 kinase regulatory subunit 2 (PIK3R2) and increases cell tight junction protein levels to relieve LPS-induced ALI [25].

In the present study, we successfully established a CLP rat model of sepsis-induced ALI (Figure 1A–G). Exosomes were isolated from the plasma of CLP rats to identify the characteristics of exosomal miRNAs profiles associated with sepsis. We have obtained approximately differentially expressed miRNAs from the exosomal miRNA sequencing results. In order to predict the functional distribution of miRNAs with the greatest level of change, the mirPath database was used to perform KEGG pathway analysis on the top 20 miRNAs with the most significant differences (Figure 1H). The color reflects the difference in miRNA function, redder color indicates the more concentrated miRNA function. The significantly increased miR-206-3p, miR-1b and miR-1-3p have a similar function which mainly focused on ECM–receptor interaction and gap junction. The miR-1-3p was the highest expressed miRNA among them, it was selected as the research object for the present study. In the pathological process of sepsis, the quality and quantity of exosomes secreted into body fluids are changed due to the immune stress. How likely is it that exosomes reach sufficient levels to convey a signal in sepsis, there is still no consensus. However, a recently published clinical research of 220 patients may provide some convincing clues. Patients with septic shock had higher plasma exosome levels (802 μg/ml, range 783–839 μg/ml) compared with healthy controls (204 μg/ml, range 199–222 μg/ml) and sepsis patients without shock (525 μg/ml, range 499–575 μg/ml; *P*<0.001). The study indicated that a higher plasma exosomes level was associated with more serious organ failure in critically ill patients with sepsis [26]. In our research, the up-regulated exosomal miR-1-3p is the result of the body’s overall stress response. We also verified an increase in exosomal miR-1-3p by RT-PCR in the plasma of CLP rats and sepsis patients (Figure 1J,K). Exosomes can be targeted in a variety of ways, including endocytosis, receptor–ligand binding mechanism, or direct fusion with recipient cell [27,28]. Many lung cells types, including epithelial cells, endothelial cells, and infiltrating macrophages, have the ability to produce as well as capture the exosomes. The endothelial cell damage caused by circulating exosomes may occur all around the body, which contribute to the multiple pathophysiology of organ dysfunction in sepsis [29]. We have observed an increased miR-1-3p in the lung tissue of CLP rat model and local tracheal LPS administration rat (Figure 1L,M). As both endotoxin and exosomes exist in the circulation during sepsis, the increase in miR-1-3p might be the co-effect of endotoxin and exosomes transport in CLP model. Considering the high expression of miR-1-3p in the plasma and lung tissue of CLP rat, and endothelial cells treated with CLP-Exo have also showed a significant increase in miR-1-3p, the results above have indirectly confirmed the role of plasma exosomes in delivering miR-1-3p into endothelial cells. Besides, substantial evidence exists for a critical effect of exosomes in the development of ALI [30], it has been verified that injection of blood exosomes from LPS-treated rats to naive rats induces ARDS [31]. Therefore, we speculated that circulating exosomes might be taken up by endothelial cells and involved in the progression of sepsis-induced lung injury through delivering miR-1-3p.

As the first encoded miRNA, miR-1-3p is associated with a series of human diseases. Studies have revealed the significant suppressor part of miR-1-3p in cancers including bladder cancer, hepatocellular carcinoma, and non-small cell lung cancer [32,33]. MiR-1-3p regulates cell proliferation, apoptosis, and invasion by targeting chemokine C–C motif ligand 2 (CCL2), protein regulator of cytokinesis 1 (PRC1) and c-mesenchymal epithelial transition factor (c-MET), also takes part in the reconstruction of pulmonary blood vessels during pulmonary hypertension through sphingosine kinase 1 (SPHK1) [34]. Vascular leakage is an important symptom of sepsis-induced ALI. The destruction of endothelial cell barrier function caused by cytoskeleton contraction and interruption of intercellular connections are the main reasons [35]. Although studies have reported that many miRNAs such as miR-486-5p and miR-146a promote cell apoptosis and amplify pulmonary inflammatory cascade to accelerate the progress of ALI [21,22], no relevant studies have explored the biofunction of miR-1-3p in pulmonary vascular endothelial cell dysfunction during sepsis.

MiR-1-3p mimics and inhibitor were transfected to up-regulate or down-regulate miR-1-3p expression in HUVECs, respectively (Figure 2C). Inhibited proliferation, increased apoptosis ratio, and active-casp3 protein together with elevated production of IL-1β and iNOS were observed in the miR-1-3p mimics group with or without LPS stimulation. However, these effects were reversed with miR-1-3p inhibitor treatment (Figure 2D–N). Previous studies have also indicated the pro-apoptotic effect of miR-1-3p in other cell types, including oral squamous cell carcinoma and human keloid fibroblasts [36,37]. The improved inflammatory response by miR-1-3p mimics (with increased IL-1β) might be beneficial to the stress response of the body, however, under the circumstance of sepsis, the enhanced inflammation are more likely to aggravate tissue damage, as exosomal miR-155 and miR-126 have been reported to induce lung inflammatory response through NF-kB and TNF-α [38,39]. Besides, inflammasome-dependent pyroptosis is responsible for the maturation and release of IL-1β, which contributes to sepsis induced lung injury [40], it is very likely that exosomal miR-1-3p might initiate IL-1β-mediated inflammation by targeting molecules on the inflammasome/pyroptosis pathway, which we will continue to study in the future. Exosomes are like a double-edged sword. The function of exosomes is related to the microenvironment of the parent cells from which they are derived. On one hand, exosomes derived from pathological conditions may mediate damage to the body. On the other hand, adipose-derived mesenchymal stem cell-derived exosomes have been reported to alleviate overwhelming systemic inflammatory reaction and organ damage and improve outcome in sepsis rat [41]. At present, the understanding of the kinetics of exosomes formation, the regulation mechanism of exosomal components, and the exosomal markers are still relatively limited, the functional heterogeneity of exosomes indicates a broad application prospects in disease diagnosis and treatment, which needs to be continue explored.

Overexpression of miR-1-3p leads to an increase in GRP78 expression, which is an ER homeostasis sensor and was regarded as a marker of ER stress together with downstream ATF4/CHOP (activating transcription factor4/C-EBP homologous protein) pathway [42]. A decreased GLP1R (Figure 4F) expression under miR-1-3p mimics was observed. Researches have confirmed that GLP1R mediates ER stress reduction through GLP1R/PI3K/Akt pathway, which also involves ATF4/CHOP and Gadd34 (growth arrest and DNA damage-inducible protein), contributes to dephosphorylation of eIF2α (eukaryote initiation factor 2 α) [43,44].

Cytoskeleton protein F-actin (fibrous actin) is a type of multifunctional protein that forms microfilaments and undergoes a rapid aggregation and disaggregation to provide mechanical support for cell morphology and movement [45,46]. The binding of F-actin to p-MLC caused cytoskeleton contraction and intercellular space formation, playing an important role in the permeability of endothelial cells [47]. The experiments above combined with Transwell and LDH detection further confirmed that miR-1-3p induces a higher permeability of vascular endothelial cells and membrane injury by regulating cytoskeleton contraction and expression of cell junction-related proteins (Figure 3F).

MiRNA binds to the 3′UTR terminal of target genes to inhibit gene transcription through incomplete complementary binding or to mediate degradation of target genes through complete complementary binding, thereby regulating target gene expression and subsequent protein synthesis [48]. The miRNA databases indicated that SERP1 is a potential target gene of miR-1-3p, which is involved in modulating cell proliferation and apoptosis through the Wnt/β-catenin or NF-kB signaling pathways [49,50]. Overexpression of SERP1 reverses miR-124-induced cell death under hypoxia in glioblastoma [51]. MiR-1-3p also demonstrated a negative regulatory effect of SERP1 mRNA or protein levels, and luciferase reporter further confirmed the binding site of SERP1 3′UTR and miR-1-3p (Figure 4E–J).

However, it remains unknown whether the effects of miR-1-3p on endothelial cell function is mediated by SERP1. Therefore, we down-regulated SERP1 by transfecting siRNA with HUVECs and found that si-SERP1 demonstrated a similar effect to miR-1-3p mimics on the function of HUVECs (Figures 5 and 6). The SERP1 plasmid was used to further strengthen the causal link between SERP1 and miR-1-3p, we found that the forced SERP1 overexpression protects against deleterious effects of miR-1-3p mimics in apoptosis, cell contraction and inflammation, except proliferation (Figure 7). The reason may be that miR-1-3p does not mainly inhibit cell proliferation through SERP1, but through other target genes such as SOX9 (sex determining region Y-box 9) [52] or STC2 (stanniocalcin 2) [53].

Taken together, we can conclude that exosomal miR-1-3p is significantly increased in CLP rat models, mediates proliferation inhibition, and increases apoptosis, cell contraction, permeability, and membrane injury of endothelial cells through its target gene SERP1, leading to vascular barrier dysfunction and participating in the occurrence of sepsis-induced pulmonary injury. This is the first time that the relationship between miR-1-3p and SERP1 has been elucidated and may provide potential new targets against sepsis-induced lung injury.

## Clinical perspectives

- Sepsis is one of the leading causes of death in ICU patients, while lung is one of the most vulnerable organs subjected to serious damage. We found that miR-1-3p was overexpressed in CLP rat plasma-derived exosomes sequencing. We investigated the effect of exosomal miR-1-3p and its target gene SERP1 on endothelial cells during sepsis.
- The present study showed that exosomal miR-1-3p is significantly increased in CLP rat models, mediates proliferation inhibition, and increases apoptosis, cell contraction, permeability, and membrane injury of endothelial cells through its target gene SERP1, leading to vascular barrier dysfunction and participating in the occurrence of sepsis-induced lung injury.
- miR-1-3p inhibitor suppressed cell apoptosis, improved permeability and membrane injury of endothelial cells. These suggest a potential therapeutic role of miR-1-3p and SERP1 in improving sepsis-induced lung injury and will be easily translated into clinical practice.

## Data Availability

The data used to support the findings of the present study are available from the corresponding author upon request.

## Abbreviations

ALI: acute lung injury
ATF4/CHOP: activating transcription factor4/C-EBP homologous protein
BSA: bovine serum albumin
CCK-8: cell counting kit-8
CLP: cecal ligation and puncture
CLP-Exo: CLP-derived exosomes
Cx43: connexin43
ER: endoplasmic reticulum
GLP1R: glucagon-like peptide 1 receptor
GRP78: glucose regulator protein 78
HUVEC: human umbilical vein endothelial cell
ICU: intensive care unit
LDH: lactate dehydrogenase
LPS: lipopolysaccharide
NC: negative control
p-FAK397: phosphorylated focal adhesion kinase
p-MLC: phosphorylated myosin light chain
RT-PCR: real-time polymerase chain reaction
SERP1: stress-associated ER protein 1
TNF: tumor necrosis factor
UTR: untranslated region

## References

[1] Rhodes A., Evans L.E., Alhazzani W., Levy M.M., Antonelli M., Ferrer R. et al. (2017) Surviving Sepsis Campaign: International Guidelines for Management of Sepsis and Septic Shock: 2016. Intensive Care Med. 43, 304–377 10.1007/s00134-017-4683-6 28101605

[2] Miyashita T., Ahmed A.K., Nakanuma S., Okamoto K., Sakai S., Kinoshita J. et al. (2016) A three-phase approach for the early identification of acute lung injury induced by severe sepsis. In Vivo (Brooklyn) 30, 341–349, http://www.ncbi.nlm.nih.gov/pubmed/27381595 27381595

[3] Sevransky J.E., Martin G.S., Shanholtz C., Mendez-Tellez P.A., Pronovost P., Brower R. et al. (2009) Mortality in sepsis versus non-sepsis induced acute lung injury. Crit. Care 13, R150 10.1186/cc8048 19758459 PMC2784371

[4] Deutschman C.S. and Tracey K.J. (2014) Sepsis: current dogma and new perspectives. Immunity 40, 463–475 10.1016/j.immuni.2014.04.001 24745331

[5] Park E.J., Appiah M.G., Myint P.K., Gaowa A., Kawamoto E. and Shimaoka M. (2019) Exosomes in sepsis and inflammatory tissue injury. Curr. Pharm. Des. 25,4486–4495 31738129 10.2174/1381612825666191116125525

[6] Théry C., Zitvogel L. and Amigorena S. (2002) Exosomes: composition, biogenesis and function. Nat Rev Immunol. 2, 569–579 10.1038/nri855.12154376 12154376

[7] Valadi H., Ekström K., Bossios A., Sjöstrand M., Lee J.J. and Lötvall J.O. (2007) Exosome-mediated transfer of mRNAs and microRNAs is a novel mechanism of genetic exchange between cells. Nat. Cell Biol. 9, 654–659 10.1038/ncb1596 17486113

[8] Bang C. and Thum T. (2012) Exosomes: new players in cell-cell communication. Int. J. Biochem. Cell Biol. 44, 2060–2064 10.1016/j.biocel.2012.08.007 22903023

[9] Bartel D.P. (2018) Metazoan microRNAs. Cell 173, 20–51 10.1016/j.cell.2018.03.006 29570994 PMC6091663

[10] Abou El-Khier N.T., Zaki M.E. and Alkasaby N.M. (2019) Study of microRNA-122 as a diagnostic biomarker of sepsis. Egypt. J. Immunol. 26, 105–116 31926500

[11] Shi Q., Fijten R.R., Spina D., Riffo Vasquez Y., Arlt V.M., Godschalk R.W. et al. (2017) Altered gene expression profiles in the lungs of benzo[a]pyrene-exposed mice in the presence of lipopolysaccharide-induced pulmonary inflammation. Toxicol. Appl. Pharmacol. 336, 8–19 10.1016/j.taap.2017.09.023 28987381 PMC5703654

[12] Jones Buie J.N., Zhou Y., Goodwin A.J., Cook J.A., Vournakis J., Demcheva M. et al. (2019) Application of deacetylated poly-N-acetyl glucosamine nanoparticles for the delivery of miR-126 for the treatment of cecal ligation and puncture-induced sepsis. Inflammation 42, 170–184 10.1007/s10753-018-0882-8 30244405 PMC6380957

[13] Fang Y., Gao F., Hao J. and Liu Z. (2017) microRNA-1246 mediates lipopolysaccharide- induced pulmonary endothelial cell apoptosis and acute lung injury by targeting angiotensin-converting enzyme 2. Am. J. Transl. Res. 9, 1287–1296, http://www.ncbi.nlm.nih.gov/pubmed/28386354 28386354 PMC5376019

[14] Meng L., Cao H., Wan C. and Jiang L. (2019) MiR-539-5p alleviates sepsis-induced acute lung injury by targeting ROCK1. Folia Histochem. Cytobiol. 57, 168–178 10.5603/FHC.a2019.0019 31825519

[15] Xiao Y., Han J., Wang Q., Mao Y., Wei M., Jia W. et al. (2017) A novel interacting protein SERP1 regulates the N-linked glycosylation and function of GLP-1 receptor in the Liver. J. Cell. Biochem. 118, 3616–3626 10.1002/jcb.26207 28597972

[16] Fleischmann C., Andr´ A., Scherag A., Adhikari N.K.J., Hartog C.S., Tsaganos T. et al. (2016) Assessment of global incidence and mortality of hospital-treated sepsis current estimates and limitations. Am. J. Respir. Crit. Care Med. 193, 259–272 10.1164/rccm.201504-0781OC 26414292

[17] Mera S., Tatulescu D., Cismaru C., Bondor C., Slavcovici A., Zanc V. et al. (2011) Multiplex cytokine profiling in patients with sepsis. APMIS 119, 155–163 10.1111/j.1600-0463.2010.02705.x 21208283

[18] Raeven P., Zipperle J. and Drechsler S. (2018) Extracellular vesicles as markers and mediators in sepsis. Theranostics 8, 3348–3365 10.7150/thno.23453 29930734 PMC6010985

[19] Chevillet J.R., Lee I., Briggs H.A., He Y. and Wang K. (2014) Issues and prospects of microRNA-based biomarkers in blood and other body fluids. Molecules 19, 6080–6105 10.3390/molecules19056080 24830712 PMC6271291

[20] Mori M.A., Ludwig R.G., Garcia-Martin R., Brandão B.B. and Kahn C.R. (2019) Extracellular miRNAs: from biomarkers to mediators of physiology and disease. Cell Metab. 30, 656–673 10.1016/j.cmet.2019.07.011 31447320 PMC6774861

[21] Bartel D.P. (2009) MicroRNAs: target recognition and regulatory functions. Cell 136, 215–233 10.1016/j.cell.2009.01.002 19167326 PMC3794896

[22] Zeng Z., Gong H., Li Y., Jie K., Ding C., Shao Q. et al. (2013) Upregulation of miR-146a contributes to the suppression of inflammatory responses in LPS-induced acute lung injury. Exp. Lung Res. 39, 275–282 10.3109/01902148.2013.808285 23848342

[23] Luo Q., Zhu J., Zhang Q., Xie J., Yi C. and Li T. (2020) MicroRNA-486-5p promotes acute lung injury via inducing inflammation and apoptosis by targeting OTUD7B. Inflammation. 43, 975–984 10.1007/s10753-020-01183-3.31940107. 31940107

[24] Wu J., Wang Y. and Li L. (2017) Functional significance of exosomes applied in sepsis: a novel approach to therapy. Biochim. Biophys. Acta Mol. Basis Dis. 1863, 292–297 10.1016/j.bbadis.2016.10.024 27989958

[25] Zhou Y., Li P., Goodwin A.J., Cook J.A., Halushka P.V., Chang E. et al. (2019) Exosomes from endothelial progenitor cells improve outcomes of the lipopolysaccharide-induced acute lung injury. Crit. Care 23, 44 10.1186/s13054-019-2339-3 30760290 PMC6373158

[26] Im Y., Yoo H., Lee J.Y., Park J., Suh G.Y. and Jeon K. (2020) Association of plasma exosomes with severity of organ failure and mortality in patients with sepsis. J. Cell. Mol. Med. 24, 9439–9445 10.1111/jcmm.15606 32639098 PMC7417686

[27] McKelvey K.J., Powell K.L., Ashton A.W., Morris J.M. and McCracken S.A. (2015) Exosomes: mechanisms of uptake. J. Circ. Biomarkers 4, 1–9 10.5772/61186 PMC5572985 28936243

[28] Svensson K.J., Christianson H.C., Wittrup A., Bourseau-Guilmain E., Lindqvist E., Svensson L.M. et al. (2013) Exosome uptake depends on ERK1/2-heat shock protein 27 signaling and lipid raft-mediated endocytosis negatively regulated by caveolin-1. J. Biol. Chem. 288, 17713–17724 10.1074/jbc.M112.445403 23653359 PMC3682571

[29] Hashemian S.M., Pourhanifeh M.H., Fadaei S., Velayati A.A., Mirzaei H. and Hamblin M.R. (2020) Non-coding RNAs and exosomes: their role in the pathogenesis of sepsis. Mol. Ther. Nucleic Acids. 21, 51–74 10.1016/j.omtn.2020.05.012 32506014 PMC7272511

[30] Gao Y. and Raj J.U. (2021) Extracellular vesicles as unique signaling messengers: role in lung diseases. Compr. Physiol. 11, 1351–1369 10.1002/cphy.c200006 33294981

[31] Li H., Meng X., Liang X., Gao Y. and Cai S. (2015) Administration of microparticles from blood of the lipopolysaccharide-treated rats serves to induce pathologic changes of acute respiratory distress syndrome. Exp. Biol. Med. (Maywood) 240,1735–41 26088862 10.1177/1535370215591830 PMC4935343

[32] Jiao D., Chen J., Li Y., Tang X., Wang J., Xu W. et al. (2018) miR-1-3p and miR-206 sensitizes HGF-induced gefitinib-resistant human lung cancer cells through inhibition of c-Met signalling and EMT. J. Cell. Mol. Med. 22, 3526–3536 10.1111/jcmm.13629 29664235 PMC6010770

[33] Shang A., Yang M., Shen F., Wang J., Wei J., Wang W. et al. (2017) MiR-1-3p suppresses the proliferation, invasion and migration of bladder cancer cells by up-regulating SFRP1 expression. Cell. Physiol. Biochem. 41, 1179–1188 10.1159/000464379 28268231

[34] Sysol J.R., Chen J., Singla S., Zhao S., Comhair S., Natarajan V. et al. (2018) Micro-RNA-1 is decreased by hypoxia and contributes to the development of pulmonary vascular remodeling via regulation of sphingosine kinase 1. Am. J. Physiol. Lung Cell. Mol. Physiol. 314, L461–L472 10.1152/ajplung.00057.2017 29167124 PMC5900352

[35] Shapiro N., Howell M.D., Bates D.W., Angus D.C., Ngo L. and Talmor D. (2006) The association of sepsis syndrome and organ dysfunction with mortality in emergency department patients with suspected infection. Ann. Emerg. Med. 48, 583.e1–590.e1 10.1016/j.annemergmed.2006.07.007 17052559

[36] Wang Z., Wang J., Chen Z., Wang K. and Shi L. (2018) MicroRNA-1-3p inhibits the proliferation and migration of oral squamous cell carcinoma cells by targeting DKK1. Biochem. Cell Biol. 96, 355–364 10.1139/bcb-2017-0015 28763625

[37] Xu M., Sun J., Yu Y., Pang Q., Lin X., Barakat M. et al. (2020) TM4SF1 involves in miR-1-3p/miR-214-5p-mediated inhibition of the migration and proliferation in keloid by regulating AKT/ERK signaling. Life Sci. 254, 117746 10.1016/j.lfs.2020.117746 32376266

[38] Su G., Ma X. and Wei H. (2020) Review article multiple biological roles of extracellular vesicles in lung injury and inflammation microenvironment.Biomed Res Int. 2020, 10.1155/2020/5608382 PMC7378585 32733944

[39] Jiang K., Yang J., Guo S., Zhao G., Wu H. and Deng G. (2019) Peripheral circulating exosome-mediated delivery of miR-155 as a novel mechanism for acute lung inflammation. Mol. Ther. 27, 1758–1771 10.1016/j.ymthe.2019.07.003 31405809 PMC6822235

[40] Grailer J.J., Canning B.A., Kalbitz M., Haggadone M.D., Rasika M., Andjelkovic A.V. et al. (2015) Critical role for the NLRP3 inflammasome during acute lung injury. J. Immunol. 192, 5974–5983 10.4049/jimmunol.1400368 PMC4061751 24795455

[41] Chang C., Sung P., Chen K., Shao P., Yang C. and Cheng B. (2018) Adipose-derived mesenchymal stem cell-derived exosomes alleviate overwhelming systemic inflammatory reaction and organ damage and improve outcome in rat sepsis syndrome. Am. J. Transl. Res. 10, 1053–1070 29736200 PMC5934566

[42] Bastida-Ruiz D., Aguilar E., Ditisheim A., Yart L. and Cohen M. (2017) Endoplasmic reticulum stress responses in placentation - a true balancing act. Placenta 57, 163–169 10.1016/j.placenta.2017.07.004 28864006

[43] Koole C., Pabreja K., Savage E.E., Wootten D., Furness S.G.B., Miller L.J. et al. (2013) Recent advances in understanding GLP-1R (glucagon-like peptide-1 receptor) function. Biochem. Soc. Trans. 41, 172–179 10.1042/BST20120236 23356279

[44] Guan G., Zhang J., Liu S., Huang W., Gong Y. and Gu X. (2019) Glucagon-like peptide- 1 attenuates endoplasmic reticulum stress-induced apoptosis in H9c2 cardiomyocytes during hypoxia/reoxygenation through the GLP-1R/PI3K/Akt pathways. Naunyn Schmiedebergs Arch. Pharmacol. 392, 715–722 10.1007/s00210-019-01625-2 30762075

[45] Pollard T.D. and Cooper J.A. (2009) Actin, a central player in cell shape and movement. Science 326, 1208–1212 10.1126/science.1175862 19965462 PMC3677050

[46] Cerutti C. and Ridley A.J. (2017) Endothelial cell-cell adhesion and signaling. Exp. Cell Res. 358, 31–38 10.1016/j.yexcr.2017.06.003 28602626 PMC5700119

[47] Thomas G.W., Rael L.T., Bar-Or R., Mains C.W., Slone D.S., Boyd S.R. et al. (2012) Biphasic effect of danazol on human vascular endothelial cell permeability and f-actin cytoskeleton dynamics. Biochem. Biophys. Res. Commun. 421, 707–712 10.1016/j.bbrc.2012.04.066 22542943

[48] Matsuyama H. and Suzuki H.I. (2020) Systems and synthetic microRNA biology: from biogenesis to disease pathogenesis. Int J Mol Sci. 21, 1–23 10.3390/ijms21010132 PMC6981965 31878193

[49] Zhang G., Song K. and Yan H. (2019) MicroRNA-124 represses wound healing by targeting SERP1 and inhibiting theWnt/β-catenin pathway. Adv. Clin. Exp. Med. 28, 711–718 10.17219/acem/94163 30740945

[50] Ma Q., Wu X., Wu J., Liang Z. and Liu T. (2017) SERP1 is a novel marker of poor prognosis in pancreatic ductal adenocarcinoma patients via anti-apoptosis and regulating SRPRB/NF-κB axis. Int. J. Oncol. 51, 1104–1114 10.3892/ijo.2017.4111 28902358 PMC5592859

[51] Mucaj V., Lee S.S., Skuli N., Giannoukos D.N., Qiu B., Eisinger- Mathason T.S.K. et al. (2015) MicroRNA-124 expression counteracts pro- survival stress responses in glioblastoma. Oncogene 34, 2204–2214 10.1038/onc.2014.168 24954504 PMC4275412

[52] Zhang H., Zhang Z., Gao L., Qiao Z., Yu M., Yu B. et al. (2019) miR-1-3p suppresses proliferation of hepatocellular carcinoma through targeting SOX9. Onco Targets Ther. 12, 2149–2157 10.2147/OTT.S197326 30962696 PMC6434909

[53] Ke J., Zhang B.-H., Li Y.-Y., Zhong M., Ma W., Xue H. et al. (2019) MiR-1-3p suppresses cell proliferation and invasion and targets STC2 in gastric cancer. Eur. Rev. Med. Pharmacol. Sci. 23, 8870–8877 31696489 10.26355/eurrev_201910_19282

